# Acute Conjunctivitis with Episcleritis and Anterior Uveitis Linked to Adiaspiromycosis and Freshwater Sponges, Amazon Region, Brazil, 2005

**DOI:** 10.3201/eid1504.081282

**Published:** 2009-04

**Authors:** Marcia O. Mendes, Mario A.P. Moraes, Ernesto I.M. Renoiner, Marta H.P. Dantas, Tatiania M. Lanzieri, Carlos F. Fonseca, Expedito J.A. Luna, Douglas L. Hatch

**Affiliations:** Ministry of Health, Brasília, Brazil (M.O. Mendes, E.I.M. Renoiner, M.H.P. Dantas, T.M. Lanzieri, E.J.A. Luna); University of Brasília, Brasília (M.A.P. Moraes); Reference Hospital of Augustinópolis, Augustinópolis, Brazil (C.F. Fonseca); Centers for Disease Control and Prevention, Atlanta, Georgia, USA (D.L. Hatch); Department of Public Health, Los Angeles, California, USA (D.L. Hatch)

**Keywords:** Conjunctivitis, scleritis, ocular nodule, anterior uveitis, adiaspiromycosis, Emmonsia sp, fresh water sponges, spicules, research

## Abstract

We conducted an epidemiologic investigation of an outbreak of ocular disease among children to determine whether the disease was linked to *Emmonsia* sp., a rarely-reported fungus and an agent of adiaspiromycosis. Using an unmatched case–control study design, we compared case-patients with asymptomatic controls randomly selected from the population. Scleral biopsies were analyzed microscopically. Of 5,084 children examined, 99 case-patients were identified; mean age (+1 SD) was 11.0 ± 4.4 years. Symptoms included photophobia (57%), ocular pain (42%), and blurred vision (40%). In the multivariate analysis, risk factors included diving in the Araguaia River (odds ratio 5.2; 95% confidence interval 2.4–12.0). Microscopy identified foreign bodies consistent with adiaconidia. This outbreak probably resulted from foreign-body–type reactions to adiaspiromycosis conidia after initial irritation caused by conjunctival contact with spicules of sponges in the river. Symptomatic children responded to corticosteroid treatment. Adiaspiromycosis is a preventable cause of ocular disease in the Amazon region.

## Abstract 2

An epidemiologic investigation of an ocular disease outbreak among children was linked to the unusual fungus *Emmonsia* sp., an agent of adiaspiromycosis.

Adiaspiromycosis, caused by the fungus *Emmonsia* sp., was first identified in Brazil during pathologic examination of lung tissue in a patient with pneumonia who died unexpectedly during treatment (*1*). Conidia of *Emmonsia* sp. are commonly present in the environment, mainly in soil and dust, and some studies have shown that pulmonary infection most often results from inhalation (*2*–*4*). Conidia, which also affect other mammals, including marsupials and rodents, do not cause infection; rather, disease is caused by the robust, multicellular immunologic response in tissue against the growing conidia, which results in noncaseating granulomas (*2*). Human pulmonary adiaspiromycosis has been reported in the literature from multiple countries, including Russia, the Czech Republic, Guatemala, Brazil, and the United States (*1*–*4*); disseminated infection may occur in immunocompromised persons. Diagnosis is most frequently made by experienced pathologists who microscopically examine tissue using various stains, including periodic acid-Shiff, which shows large, round, multiwalled structures with surrounding foreign-body–type mixed cellular reactions (*3*–*5*).

On October 26, 2006, local ophthalmologists notified the State Health Secretariat in Tocantins, located in the northern Amazon region of Brazil, of an unusual outbreak of conjunctivitis with ocular nodules of unknown etiology among children. Illness was identified in 17 children, 16 of whom were <15 years of age; all were residents of Araguatins (population = 29,336), a city located along the slow-moving Araguaia River. The disease had remained underdiagnosed and underreported in Araguatins until an ophthalmologic service was initiated in the neighboring city of Augustinópolis, where the initial case-patients were referred, examined, and subsequently reported to local health authorities. Shortly after the condition was reported to Brazil’s Ministry of Health, a team of epidemiologists, laboratorians, and ophthalmologists began an investigation with the following objectives: 1) determine the magnitude of the problem, 2) identify the cause, and 3) implement prevention and control activities.

## Methods

### Screening

Because 16 (94%) of the 17 initial case-patients reported were 5–15 years of age, active searches were conducted in 40 of the 41 primary schools in Araguatins. Health workers were trained by ophthalmologists to identify children with clinical signs that were similar to the initial 17 case reports. Children with blurred vision, or any of the following, were referred for an ophthalmologic examination: conjunctival injection or inflammation, nodules on conjunctiva or sclera, or cornea with any discoloration or opacification.

###  History

We defined a case of confirmed ocular disease (COD) in a child with any of the following physical signs: conjunctival injection or inflammation, nodules on sclera, or conjunctival or corneal opacities with anterior uveitis identified during ophthalmologic examination (including by slit-lamp and microscopy). Patients with COD were interviewed by using a standardized semistructured questionnaire; parents served as proxies for young children. Information was collected about basic demographic characteristics, duration and type(s) of symptoms, source of drinking water, frequency and specific locations where children had exposure to the local freshwater river, and similar illness in family members.

### Case–Control Study

We hypothesized that exposure to the Araguaia River played a role in the chain of events resulting in ocular disease. This hypothesis was tested by using an unmatched case–control study design, based on an estimated 90% of case-patients having prior ocular exposure to river water; and by using 80% power, an α level of 0.05, and a case:control ratio of 1:3, which yielded a study sample size of 62 case-patients and 186 controls. The 62 case-patients included in this study were randomly selected from a total of 91 children with COD identified and interviewed. Two separate control groups were selected for interviews. The first group (community controls) included 186 asymptomatic persons ranging from 5 to 20 years of age living in households systematically selected from randomly chosen residential blocks in the urban area of Araguatins municipality. A second control group (household controls) comprised all asymptomatic residents of case-patient households.

#### Statistical Analysis

In the univariate analysis of the case–control study data, categorical variables were tested by using a χ^2^ test, and continuous variables were compared by using a Kruskall-Wallis or *t* test, as appropriate. The odds ratio (OR) was used as the measure of association, 95% confidence intervals (CIs) were calculated, and p<0.05 was considered significant. Using a stepwise backward elimination strategy to calculate the adjusted OR, an unconditional logistic regression model was used for the multivariate analysis.

#### Laboratory Methods

Serologic tests from children with COD included ELISA tests for onchocercosis (immunoglobulin [Ig] G), toxoplasmosis (IgM), and toxocariasis (IgG). Blood smears and aqueous humor from selected patients were examined microscopically for evidence of microfilaria. Biopsy samples from COD case-patients with scleral nodules or corneal abnormalities were fixed in formalin, stained with hemotoxylin and eosin, and periodic acid-Schiff, and examined microscopically. Soil samples were examined for helminth eggs and larvae. Water samples were collected from areas of the Araguaia River where case-patients reportedly swam. These samples were examined for 1) freshwater sponges, which were identified to species, and 2) silicious spicules (gemmoscleres) of these sponges; details of the methods and results of this sampling have been published (*6*,*7*).

## Results

In addition to the initial 17 COD case-patients who were examined by ophthalmologists and reported to the Ministry of Health, a total of 5,084 children 5–15 years of age (corresponding to 83% of this age group in the population) were examined at 40 schools by health workers. During these active searches, of 235 students triaged and referred for evaluation of possible ocular abnormalities, 64 (27%) were categorized by ophthalmologists as having COD and 103 (44%) had sequelae. In addition to the total 81 COD case-patients identified above by November 26, 2005, COD was diagnosed for an additional 18 by January 26, 2006, identified initially by local clinicians or through patient self-referral.

**Figure 1:**
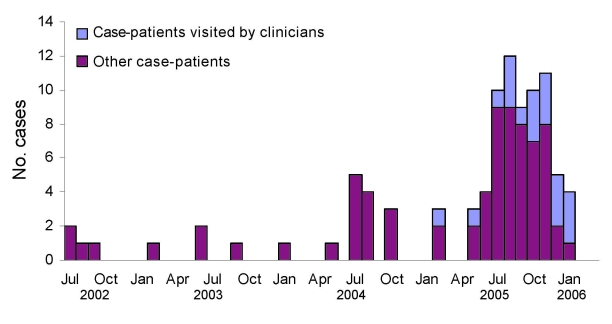
Date of symptom onset for 84 patients in whom confirmed ocular infection was diagnosed, Araguatins, Brazil, 2002–2005.

Of the 99 COD case-patients identified, 91 (92%) were interviewed, of whom 70 (77%) were male and the mean age ( ±1 SD) was 11.0 ± 4.4 years. Ocular-related signs and symptoms were conjunctival hyperemia (89%), conjunctival nodule (70%), excessive tearing (63%), conjunctival pruritus (60%), photophobia (57%), ocular pain (42%), and blurred vision (40%); other reported symptoms included headache (37%) and generalized pruritus (16%). The number of COD case-patients peaked during the dry season (July–September) (Figure 1) when schools were closed and contact with the local river was most common. Of those interviewed, 88 (97%) resided in urban areas of Araguatins, and 3 (3%) resided in rural areas.

**Figure 2:**
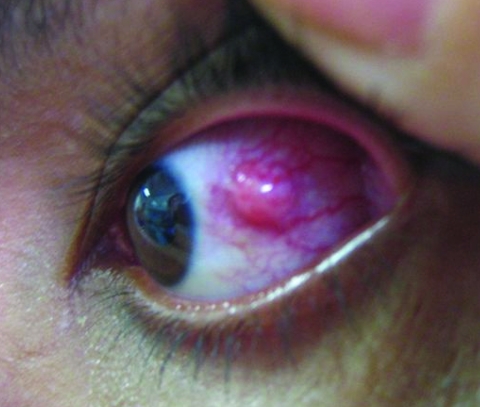
Conjunctival infection and opaque scleral nodule with vascularization in case-patient with confirmed ocular disease, Araguatins, Brazil. Source: Dr Leandro Alencar/Dr Carlos Franklin.

**Figure 3:**
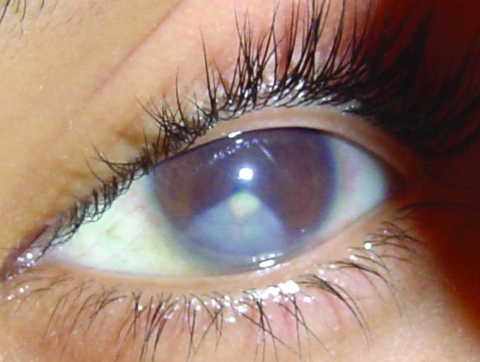
Diffuse opacification of lower quadrant of anterior chamber and cornea with anterior sinéquia in case-patient with confirmed ocular disease Araguatins, Brazil. Source: Dr Leandro Alencar/Dr Carlos Franklin.

Ophthalmologists identified unilateral ocular lesions in 73 (80%) of interviewed COD case-patients; in 18 (20%) patients, the lesions were bilateral. Unilateral subconjunctival nodule(s) of sclera (Figure 2), some of which extended to the corneal limbus, were identified in 43 (47%) case-patients and were present bilaterally in 12 (13%). Unilateral corneal opacities (Figure 3) were observed in 32 (35%) case-patients, bilaterally in 18 (20%).

Neighborhoods most commonly affected in the city of Araguatins included Centro, where 61 (67%) of all case-patients resided (prevalence of 8 cases per 100,000 inhabitants), Vila Cidinha (6 per 100,000), and Nova Araguatins (3 per 100,000). Seventy-five (82%) case-patients attended an urban school in Araguatins, and 3 (3%) attended a rural school (Table 1); 13 (14%) of case-patients were not enrolled in a school.

**Table 1:** Prevalence of confirmed ocular disease in schools surveyed, stratified by urban area, Araguatins, Brazil, 2005

School zone*	No. case-patients identified	No. students	Prevalence, %
Urban	75	3,299	2.3
A	11	179	6.2
B	28	664	4.2
C	14	615	2.3
D	9	379	2.4
E	1	88	1.4
F	4	331	1.2
G	4	331	1.2
H	4	581	0.7
Rural	3	1,785	0.2
I	1	26	3.8
J	1	52	1.9
L	1	108	0.9

*Area where schools are located.

Of the 62 COD case-patients randomly selected to participate in the case–control study, 48 (77%) were male. Case-patients were significantly younger (mean 11.4 ± >3.5 years) than household controls (mean 25 ± 17.8 years; p<0.001, Student *t* test), but age distribution was similar to that of community controls (13 ± 6.0 years; p = 0.4, Student *t* test). In univariate analysis, male sex was significantly associated with disease when case-patients were compared with 178 household controls (OR 4.7, 95% CI 2.3–9.8, p<0.001); and with 186 community controls (OR 4.5, 95% CI 2.2–9.4, p<0.001).

Environmental exposures most strongly associated with increased risk for disease, which was significant when compared with both household and community controls (Table 2), were swimming or diving in the Araguaia River and frequenting Cais beach on the bank of the Araguaia River. Fishing in the river was associated with disease but only when case-patients were compared with community controls. Factors not significantly associated with disease (using either control group) were drinking untreated river water, washing clothes in the river, contact with various types of domesticated animals, a history of exposure to ticks, or a history of allergies. Frequency of river contact was also significantly associated with disease (Table 3). According to multivariate analysis, factors most strongly associated with disease were being of male sex, frequenting the Cais beach area, and diving underwater in the Araguaia River (Table 4).

**Table 2:** Results of univariate analysis of case–control study on relationship between various exposures to freshwater rivers and confirmed ocular disease, Araguatins, Brazil, 2005*

Exposure	No. (%) case-patients exposed, n = 62	Household control group, n = 178				Community control group, n = 186		
		No. (%) exposed	OR	95% CI		No. (%) exposed	OR	95% CI
Contacting river water	61 (98)	132 (74)	21.3	3.0–424.2		145 (78)	17.2	2.5–344.8
Diving underwater	49 (83)	67 (38)	8.1	3.7–18.4		90 (49)	5.1	2.3–11.5
Visiting Cais Beach	50 (81)	97 (55)	3.4	1.6–7.2		52 (30)	10.1	4.7–21.9
Fishing	30 (48)	74 (42)	1.3	0.7–2.4		54 (29)	2.3	1.2–4.3
Drinking untreated river water	23 (38)	62 (35)	1.1	0.6–2.2		50 (27)	1.7	0.9–3.3
Washing clothes in river water	6 (10)	43 (24)	0.3	0.1–0.9		29 (16)	0.6	0.2–1.6

*OR, odds ratio; CI, confidence interval.

**Table 3:** Results of univariate analysis of case–control study on relationship between frequency of exposure to Araguaia River and odds of confirmed ocular disease, Araguatins, Brazil, 2005*

Frequency	No. (%) case-patients exposed, n = 62	Household control group, n = 178			Community control group, n = 186	
		No. (%) exposed	OR†		No. (%) exposed	OR‡
Weekly	25 (40)	20 (11)	6.9		13 (7)	10.8
Once every 2 weeks	4 (7)	5 (3)	4.4		7 (4)	3.2
Vacations	25 (40)	109 (61)	1.3		121 (65)	1.2
Rarely§	8 (13)	44 (25)	1.0		45 (24)	1.0

*OR, odds ratio. †χ^2^ test for trend 25.7; p<0.0001. ‡χ^2^ test for trend 35.3; p<0.0001. §Fewer than 3×/year.

**Table 4:** Results of multivariate analysis of case–control data showing independent effect of type of exposures to Araguaia River and risk for confirmed ocular disease, Araguatins, Brazil, 2005*

Exposure to Araguaia River	Household control group			Community control group	
	aOR (95% CI)	p value		aOR (95% CI)	p value
Swimming	3.1 (0.4–26.8)	0.3		2.1 (0.2–19.4)	0.5
Diving underwater	4.6 (1.9–10.6)	0.0004		2.7 (1.1–7.1)	0.04
Visited Cais Beach	3.2 (1.4–7.1)	0.005		9.9 (4.3–22.9)	0.00001
Male gender	3.4 (1.6–7.2)	0.001		4.7 (1.9–11.0)	0.0004
Fishing in river	–	–		1.2 (0.5–2.7)	0.6

*Unconditional logistic regression model used. aOR, adjusted odds ratio; CI, confidence interval.

Among 32 case-patients treated with corticosteroid (oral and/or topical prednisone) by ophthalmologists, disease was resolved or cured in 25 (78%); 7 (22%) case-patients had more severe symptoms and were referred to the Sao Geraldo Hospital in Belo Horizonte, Minas Gerais State.

**Figure 4:**
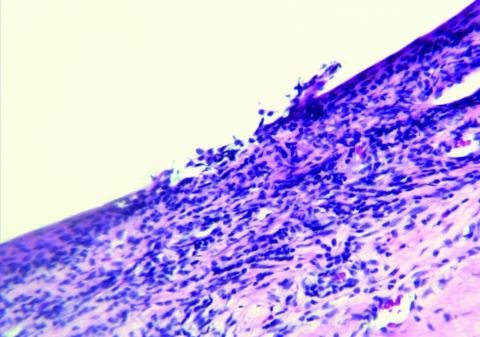
Scleral nodule biopsy sample, showing microulceration of corneal epithelium (magnification ×20, hematoxylin and eosin stain), Araguatins, Brazil. Source: Department of Pathology, University of Brasília.

**Figure 5:**
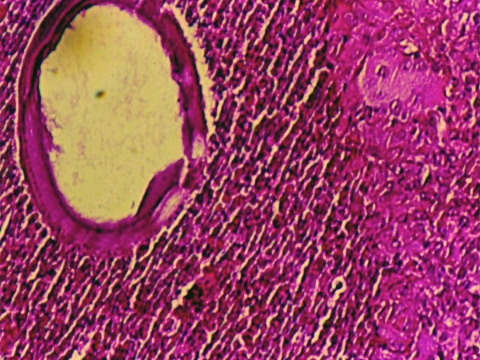
Scleral nodule biopsy specimen, showing diffuse, subconjunctival mixed-cellular infiltrate surrounding large, thick-walled adiaconidia of *Emmonsia* sp. (magnification ×200, hematoxylin and eosin stain), Araguatins, Brazil. Source: Department of Pathology, University of Brasília.

Among those with nodules, 14 had biopsy samples taken under sterile surgical conditions for diagnostic purposes. Microscopic examination of nodules identified microulcerations of corneal epithelium (Figure 4), and a mixed acute inflammatory response mainly consisting of leukocytes, with some eosinophils, and lymphohistocytic and diffuse neutrophilic infiltrates with edema. Twelve case-patients (13%) had a granuloma of the anterior chamber of the eye unilaterally; 1 case-patient had bilateral anterior chamber granulomas. In addition, in 2 biopsy samples, subconjunctival inflammation was present surrounding large, 200–600-micron, thick-walled, spherical foreign bodies (Figure 5) consistent with adiaconidia of *Emmonsia* sp. fungus, a cause of adiaspiromycosis.

 Onchocerciasis, toxoplasmosis, toxocariasis, and microfilaria were discarded as possible etiologies for COD. All 17 samples tested for onchocerciasis were nonreactive for IgG, and no evidence of microfilaria was found in blood smears (n = 17), aqueous humor (n = 1), or biopsy samples of cutaneous nodules (n = 6) examined microscopically. All samples tested for toxoplasmosis (n = 46) were nonreactive for IgM antibodies. Serologic tests for detection of IgG for toxocariasis were reactive for 59 (88%) of 67 COD case-patients, 14 (74%) of 19 household controls, and 53 (64%) of 82 community controls; helminth eggs and larvae, including *Toxocara* spp., were found in 7 of 10 soil samples.

## Discussion

These results confirm the existence of an outbreak of conjunctivitis and severe ocular disease probably caused by adiaspiromycosis, mainly among school-aged children. Risk factors for COD identified in this investigation included diving underwater and frequenting a specific beach (Cais) on the Araguia River in the Amazon region of Brazil. The precise reasons eye contact with river water increased risk remain unclear. Perhaps exposure to freshwater sponge spicules caused an initial conjunctival irritation, as suggested in previous publications (*6*–*8*). However, the microscopic identification of probable adiaconidia of *Emmonsia* sp. fungus in the scleral biopsy samples from children with severe disease in this outbreak suggests that conjunctival irritation was most likely followed by conjunctival exposure to conidia of this fungus, perhaps in dust caused in part by dry environmental conditions, similar to the exposure of respiratory mucosa described in case reports of pulmonary adiaspiromycosis (*1*–*5*,*9*). Adiaspiromycosis causes an inflammatory and often granulomatous response in tissue because of the presence of nonbudding, thick-walled adiaconidia of *Emmonsia* sp. fungus. Disease is thought to result from exposure to conidia (through inhalation or mucosal contact with dust); these conidia subsequently cause a marked inflammatory response and enlarge to become adiaconidia ranging in diameter from 300 to 600 microns (*1*–*5*,*9*,*10*).

Boys were at higher risk than girls most likely because boys had more facial and eye contact with the river water while swimming and diving. To minimize bias, we randomly selected asymptomatic controls among persons 5–25 years of age in the community, but some selection bias may have resulted because boys and adolescents were absent at the time of interview (only 42% of community-based controls were boys). The clinical characteristics of conjunctivitis in this outbreak were unusual for several reasons. First, unlike conjunctivitis caused by common bacterial or viral pathogens, neither purulent conjunctival discharge nor hemorrhage was reported, and family members of case-patient households were not commonly affected. In addition, disease was characterized by unusual, single or multiple, white, opaque scleral nodules, often with hyperemia or local edema, and in some cases with opacification (changes in the normally transparent characteristics of the cornea or superficially on scleral tissue) extending to the limbus, or angular corneal opacities and anterior uveitis with granulomas in the anterior chamber. We believe that the clinical improvement of nearly all patients treated with corticosteroids also argues strongly against a bacterial cause or fungal species other than *Emmonsia* because conidia of *Emmonsia* sp. enlarge and cause a localized inflammatory response but do not commonly have the potential to disseminate.

Characteristics of this outbreak are similar to those of an outbreak of anterior uveitis and granuloma previously reported in India, where the etiology was traced to trematodes (*10*). Although the thick-walled foreign body observed microscopically on slides from 2 case-patients was initially suspected to be trematodes, the round, apparently spherical shape, thick walls, and vacuous central area with lack of organized, internal structures is most consistent with the adiaconidia of the *Emmonsia* sp. Morphologic appearance differs from that of the fungus *Coccidiodes immitis,* in which spores contain internal microsporules (*11*).

The natural history of this disease is unknown. However, we identified COD case-patients in several stages of disease, including patients with sequalae. Moreover, after obtaining school surveys, we identified ≈5% of children with ocular abnormalities; COD was diagnosed in one third of children after an ophthalmologic exam. We educated the population about risks for eye contact with river water; active searches were conducted to identify all ill persons in the population and in neighboring cities, and health officials limited recreational access to the Araguaia River. These findings suggest that the extent of this problem may be more widespread in the Amazon region than is currently recognized.
